# The Lipophilic Extract from *Ginkgo biloba* L. Leaves Promotes Glucose Uptake and Alleviates Palmitate-Induced Insulin Resistance in C2C12 Myotubes

**DOI:** 10.3390/molecules29071605

**Published:** 2024-04-03

**Authors:** Tiantian Li, Quanhe Lv, Chunhui Liu, Chunfei Li, Xiaomin Xie, Wen Zhang

**Affiliations:** 1 School of Life Sciences, East China Normal University, 500 Dongchuan Road, Shanghai 200241, China; 2 China National Institute of Standardization, 4 Zhichun Road, Beijing 100191, China

**Keywords:** *Ginkgo biloba* L., lipophilic extract, C2C12 myotubes, glucose uptake, insulin resistance

## Abstract

*Ginkgo biloba* L. (ginkgo) is a widely used medicinal plant around the world. Its leaves, which have been used as a traditional Chinese medicine, are rich in various bioactive components. However, most of the research and applications of ginkgo leaves have focused on terpene trilactones and flavonol glycosides, thereby overlooking the other active components. In this study, a lipophilic extract (GL) was isolated from ginkgo leaves. This extract is abundant in lipids and lipid-like molecules. Then, its effect and potential mechanism on glucose uptake and insulin resistance in C2C12 myotubes were investigated. The results showed that GL significantly enhanced the translocation of GLUT4 to the plasma membrane, which subsequently promoted glucose uptake. Meanwhile, it increased the phosphorylation of AMP-activated protein kinase (AMPK) and its downstream targets. Both knockdown of AMPK with siRNA and inhibition with AMPK inhibitor compound C reversed these effects. Additionally, GL ameliorated palmitate-induced insulin resistance by enhancing insulin-stimulated glucose uptake, increasing the phosphorylation of protein kinase B (PKB/AKT), and restoring the translocation of GLUT4 from the cytoplasm to the membrane. However, pretreatment with compound C abolished these beneficial effects of GL. In conclusion, GL enhances basal glucose uptake in C2C12 myotubes and improves insulin sensitivity in palmitate-induced insulin resistant myotubes through the AMPK pathway.

## 1. Introduction

Diabetes mellitus is a metabolic disorder characterized by elevated blood glucose levels or hyperglycemia due to abnormalities in either insulin secretion or insulin action or both [1]. Diabetes mellitus is a chronic disease that affects the body’s ability to regulate blood glucose levels. It is characterized by high levels of glucose in the blood, which can cause damage to the heart, blood vessels, eyes, kidneys, and nerves over time [2]. The World Health Organization reports that over 90% of diabetes cases are Type 2 diabetes mellitus, also known as noninsulin-dependent diabetes mellitus or adult-onset diabetes [1,2]. The International Diabetes Federation reports that in 2019, diabetes affected 463 million adults aged between 20 and 79 years old and caused 4.2 million deaths. This number is projected to rise to 700 million by 2045 [2]. This form of diabetes originates from two primary issues related to insulin: insulin resistance and dysfunction of β-cells [1,2]. Insulin resistance is a consequence of various cellular pathway disruptions, leading to a reduced responsiveness of peripheral tissue cells, especially those in the muscle, liver, and adipose tissue, to insulin [1,2,3]. In the early stages of type 2 diabetes mellitus, the body compensates for decreased insulin sensitivity by increasing insulin secretion. However, over time, β-cell function declines and insulin deficiency develops, leading to high blood glucose levels [1]. 

Skeletal muscle plays a vital role in blood glucose regulation, absorbing glucose through both insulin-dependent and insulin-independent pathways [4]. Typically, skeletal muscle is the primary site for glucose transport and utilization. However, in states of insulin resistance, the muscle’s ability to uptake glucose in response to insulin is significantly reduced, leading to persistently high blood glucose levels and potentially resulting in metabolic disorders such as type 2 diabetes [5,6]. As the main site of insulin-stimulated glucose uptake, skeletal muscle is often considered the primary cause of whole-body insulin resistance [4,7]. Insulin resistance in skeletal muscles can occur decades before the development of β-cell failure and the onset of type 2 diabetes symptoms [4,7]. Addressing insulin resistance in skeletal muscle can restore whole-body glucose homeostasis [7,8], making the promotion of glucose uptake and insulin sensitivity in skeletal muscles crucial in preventing or reducing insulin resistance, hyperglycemia, and type 2 diabetes. Although type 2 diabetes mellitus cannot be cured, it can be effectively managed. The critical goal for patients with type 2 diabetes is to maintain glucose levels close to normal [9]. Lifestyle changes and pharmacologic interventions, including weight loss, physical activity, adherence to a Mediterranean diet, and the use of hypoglycemic agents, have been proven to prevent or delay type 2 diabetes [10]. Traditional plant-based medicinal formulations and their active constituents are globally used as a treatment for diabetes and its complications [9]. Several traditional medicines and herbs are known to delay the development of diabetes-related complications and improve metabolic abnormalities [9].

*Ginkgo biloba* L., also known as ginkgo, is the sole survivor of the Ginkgophyta division and is often referred to as a living fossil because it has been around for more than 180 million years. The leaves of ginkgo are used in traditional Chinese medicine and their usage has been documented in renowned Chinese herbal texts like Shen Nong Ben Cao Jing (2800 BC) and Pen Ts’ao Kang Mu (1596) [11]. Ginkgo leaves contain high levels of bioactive compounds, such as terpene trilactones, flavonoids, proanthocyanidins, alkylphenols, carboxylic acids, sterols, polyprenols, and so on [11]. However, most of the research and applications of ginkgo leaves have focused on terpene trilactones and flavonoids, and other active components have less been studied and utilized [12]. An untargeted LC–MS metabolomics analysis was performed on ginkgo leaves and seeds at two developmental stages, identifying 8146 known metabolites. The primary constituents of the metabolites were 29.65% lipids and lipid-like molecules, followed by 10.19% phenylpropanoids and polyketides, 7.45% organoheterocyclic compounds, 7.33% organic acids and derivatives, 5.81% organic oxygen compounds, and 4.31% benzenoids [13]. Furthermore, it was discovered that the nonsaponifiable lipids of ginkgo leaves contained terpenoids, polyprenols, sterols, and chainlike alcohols (ketone, ester) among others [11]. These studies underscore the fact that lipids and lipid-like molecules, which contain active components, are the main constituents in ginkgo leaves.

In our previous study, we found that a lipophilic extract from ginkgo leaves (GL) significantly enhanced both glucose consumption and insulin-induced glucose consumption in C2C12 myotubes. These findings suggest that GL may have a potential in improving glucose uptake, subsequently accelerating glucose utilization in muscle; meanwhile, it also may promote insulin sensitivity. Therefore, in the present study, we aimed to investigate whether GL also improves basal glucose uptake and insulin resistance in C2C12 myotubes, and to explore the potential mechanism.

## 2. Results

### 2.1. GL Promoted Glucose Consumption, Glucose Uptake, and GLUT4 Translocation in C2C12 Myotubes

**Figure 1:**
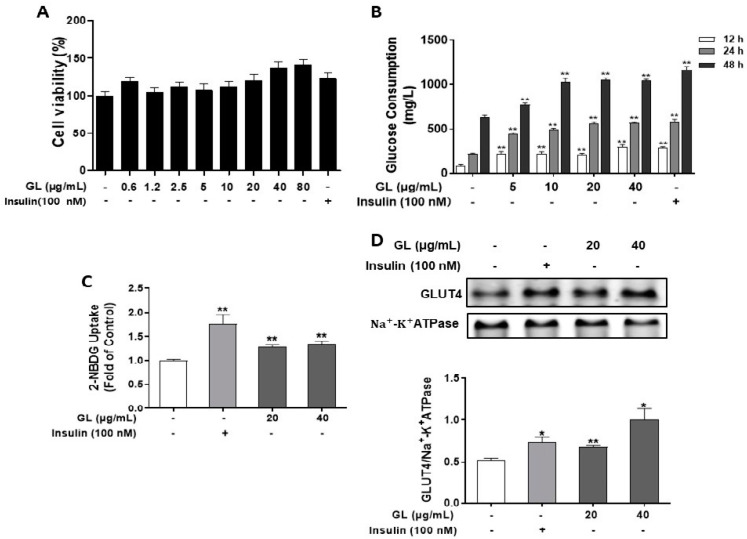
GL promoted glucose uptake and GLUT4 translocation in C2C12 myotubes. (**A**) Effect of GL on cell viability in C2C12 myotubes. The cell viability was measured by MTT assay (*n* = 6). (**B**) GL stimulated glucose consumption after treatment for 12, 24, or 48 h (*n* = 5). (**C**) GL promoted glucose uptake after 4 h treatment. Glucose uptake was measured using the 2-NBDG method (*n* = 5). (**D**) GL promoted GLUT4 level in cell membrane protein fractions (*n* = 3). All data are presented as the mean ± SD. * and ** indicate *p* < 0.05 and *p* < 0.01 compared to control, respectively.

The potential cytotoxicity of GL on C2C12 myotubes was evaluated by MTT assay. As shown in Figure 1A, treatment of 0.6–80 μg/mL GL with differentiated C2C12 myotubes for 24 h did not inhibit cell viability. The effects of GL on glucose consumption in C2C12 myotubes was evaluated. As per results shown in Figure 1B, GL concentration-dependently increased glucose consumption of myotubes. Treatment of myotubes with 5, 10, 20, and 40 μg/mL GL for 12, 24, and 48 h significantly increased glucose consumption compared with control myotubes (*p* < 0.01). Next, a 2-NBDG uptake assay was used to determine the effect of GL on glucose uptake. The results showed that 20 and 40 μg/mL GL significantly promoted glucose uptake in C2C12 myotubes by 1.28- and 1.34-fold, respectively, compared with controls (*p* < 0.01, Figure 1C). Moreover, the level of GLUT4 protein translocated to the plasma membrane was significantly increased by 1.90-fold compared with those of control myotubes (Figure 1D, *p* < 0.01). These results indicated that GL significantly stimulated GLUT4 translocation to membrane and subsequently promoted glucose uptake in C2C12 myotubes.

### 2.2. GL Promotes GLUT4 Translocation and Glucose Uptake via AMPK Pathway in C2C12 Myotubes

**Figure 2:**
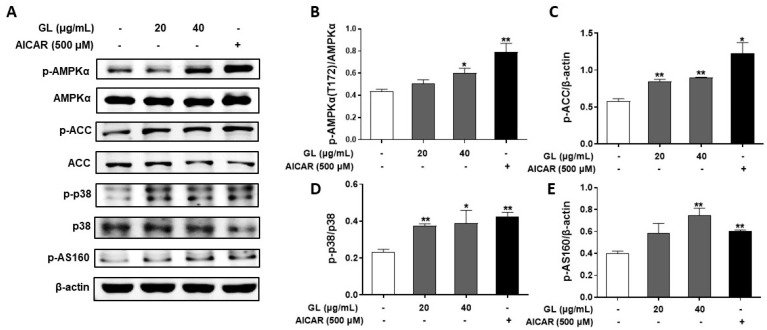
GL activated AMPK pathway in C2C12 myotubes. Fully differentiated C2C12 myotubes underwent treatment with GL for 4 h or were exposed to AICAR (500 μM) for 1 h. Western blotting analysis (**A**) and subsequent quantification (**B**–**E**) of the phosphorylation levels of AMPK, ACC, AS160, and p38 MAPK in myotubes. All data are presented as the mean ± SD (*n* = 3 or 4). * and ** indicate *p* < 0.05 and *p* < 0.01 compared to control, respectively.

To investigate the molecular basis for the promotion of glucose uptake by GL, phosphorylated levels of proteins involved in AMPK signaling pathways were examined using Western blotting analysis. As per results shown in Figure 2, exposing C2C12 myotubes to GL (40 μg/mL) for a duration of 4 h led to a significant elevation in the levels of phosphorylated AMPK, acetyl-CoA carboxylase (ACC), p38 mitogen-activated protein kinase (p38 MAPK), and AKT substrate of 160 kDa (AS160) (Figure 2A–E). These levels were increased by 1.38-, 1.56-, 1.67-, and 1.86-fold, respectively, in comparison to the control group (*p* < 0.05 or 0.01). These results indicated that GL activated the AMPK pathway.

**Figure 3:**
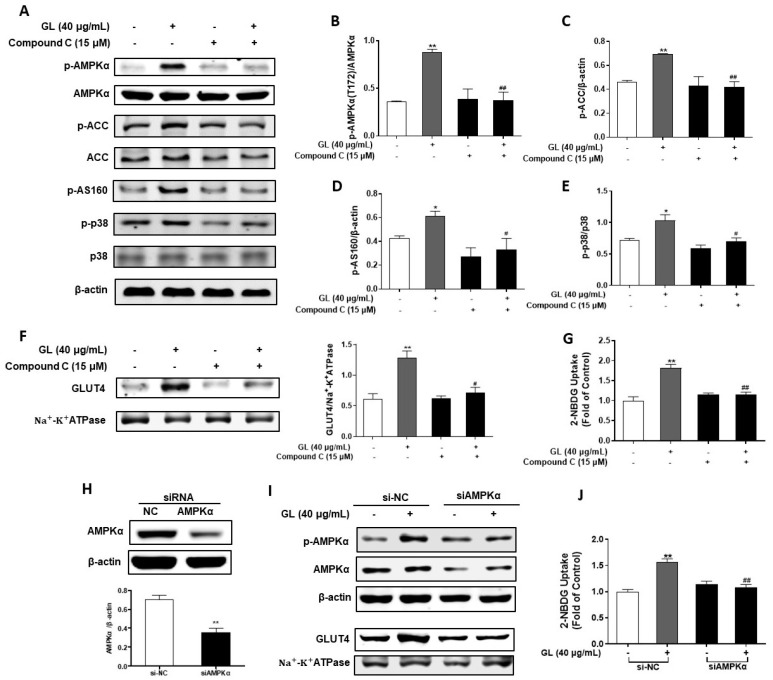
Effects of AMPK inhibitor compound C and AMPK siRNA on the glucose uptake and GLUT4 translocation stimulated by GL. Western blotting analysis (**A**) and quantification (**B**–**E**) of the phosphorylation levels of AMPK, ACC, AS160, and p38 MAPK in whole cell lysates (*n* = 3). (**F**) Western blotting and quantification of GLUT4 in cell membrane proteins (*n* = 3). (**G**) 2-NBDG assay of glucose uptake (*n* = 6). (**H**–**J**) Further analysis of AMPK activation, GLUT4 translocation, and glucose uptake (*n* = 5) in AMPK siRNA transfected C2C12 myotubes. All data are presented as mean ± SD. * and ** indicate *p* < 0.05 and *p* < 0.01 compared to control, respectively. # and ## denote *p* < 0.05 and *p* < 0.01 compared to GL group, respectively.

To confirm whether the stimulatory effect of GL on glucose uptake was mediated through AMPK activation, we pretreated myotubes with the AMPK-specific inhibitor compound C (15 μM) for 1 h, followed by treatment with GL (40 μg/mL). As per results shown in Figure 3, compound C blocked the increase in phosphorylation of AMPK, ACC, p38 MAPK, and AS160 induced by GL (Figure 3A–E, *p* < 0.05 vs. GL treatment alone). Moreover, compound C also blocked GL-induced GLUT4 translocation and glucose uptake (Figure 3F,G; *p* < 0.05 vs. GL treatment alone). 

To further confirm the significance of AMPK-dependent, AMPK siRNA (60 nM) was utilized to reduce AMPK expression in C2C12 myotubes. The expression of AMPK in C2C12 myotubes was diminished by 51% following transfection with AMPK siRNA (Figure 3H), and GL-induced increase in phosphorylation of AMPK was abolished by AMPK knockdown (Figure 3I). Consistently, AMPK knockdown blunted promoting effects of GL on GLUT4 translocation and glucose uptake in transfected C2C12 myotubes (Figure 3I,J, *p* < 0.05 vs. GL treatment alone). These results confirmed that GL significantly stimulated GLUT4 translocation and glucose uptake through the AMPK-p38 MAPK-AS160 pathway.

### 2.3. GL Activates AMPK Pathway via CaMKKβ in C2C12 Myotubes

**Figure 4:**
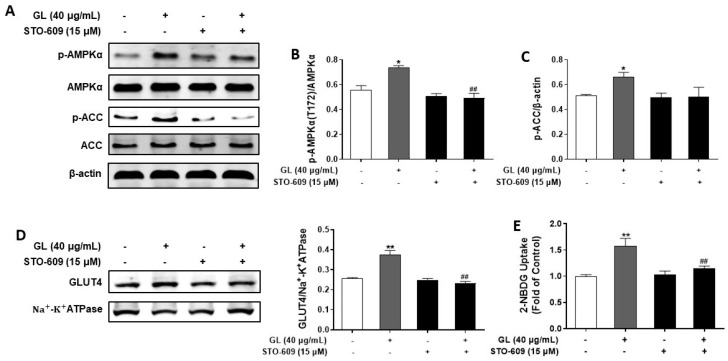
Effects of CaMKKβ inhibitor STO-609 on glucose uptake and GLUT4 translocation stimulated by GL. (**A**) Western blotting analysis and (**B**,**C**) quantification of the phosphorylation levels of AMPK and ACC (*n* = 3). (**D**) Western blotting analysis and quantification of GLUT4 in cell membrane proteins (*n* = 3). (**E**) 2-NBDG assay of glucose uptake (*n* = 6). All data are presented as mean ± SD. * and ** indicate *p* < 0.05 and *p* < 0.01 compared to control, respectively. ## denote *p* < 0.01 compared to GL group.

Studies have revealed that CaMKKβ serves as a kinase upstream of AMPK, playing a role in the activation of glucose uptake [14]. To explore the potential involvement of CaMKKβ in glucose uptake mediated by GL, we examined the effect of STO-609, an inhibitor specific to CaMKKβ, on the activation of AMPK, glucose uptake, and GLUT4 translocation induced by GL. As depicted in Figure 4A–C, the phosphorylation of AMPK and its downstream protein ACC, triggered by GL, was obstructed by STO-609 (*p* < 0.05 vs. GL treatment alone), suggesting that the AMPK pathway was activated by GL via CaMKKβ. Moreover, the translocation of GLUT4 and glucose uptake induced by GL were impeded when pretreated with STO-609 (Figure 4D,E; *p* < 0.01 vs. GL treatment alone). These findings suggest that the uptake of glucose is promoted by GL through the CaMKKβ-AMPK pathway.

### 2.4. GL Protected C2C12 Myotubes from Palmitate-Induced Insulin Resistance

**Figure 5:**
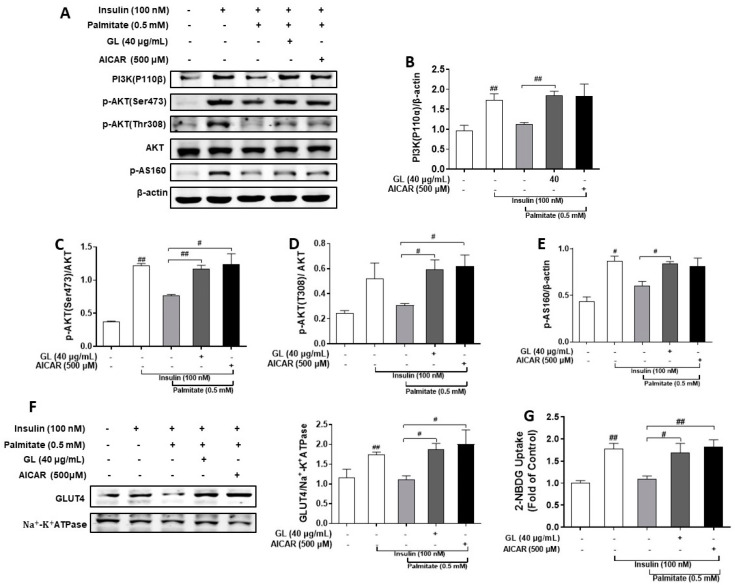
GL alleviated palmitate-induced insulin resistance in C2C12 myotubes. (**A**) Western blotting and quantification (**B**–**E**) of PI3K, phospho-AKT, and phospho-AS160 (*n* = 3). (**F**) Western blotting and quantification of GLUT4 in cell membrane proteins (*n* = 3). (**G**) 2-NBDG assay of glucose uptake (*n* = 5). All values are presented as means ± SD. # and ## denote *p* < 0.05 and *p* < 0.01 compared to model (insulin+ palmitate) group, respectively.

In the present study, we used palmitate to induce insulin resistance in C2C12 myotubes. Our results showed that treatment with 500 μM palmitate for 18 h significantly inhibited the insulin pathway. Specifically, insulin-stimulated expression of PI3K (P110β) and phosphorylation of AKT and AS160 (Figure 5A–E), as well as GLUT4 translation (Figure 5F) and glucose uptake (Figure 5G), were all significantly decreased by palmitate treatment (*p* < 0.05 or 0.01).

To test whether GL could protect against insulin resistance, we co-treated C2C12 myotubes with 40 μg/mL GL and palmitate for 18 h. We found that GL reversed the inhibitory effects of palmitate on insulin-stimulated phosphorylation of AKT and AS160 and PI3K (P110β) expression (*p* < 0.05 or 0.01, Figure 5A–E). Moreover, GL restored the palmitate-induced reduction in insulin-stimulated glucose uptake by 1.78-fold (*p* < 0.05, Figure 5G) and GLUT4 translocation to the plasma membrane (*p* < 0.05, Figure 5F), demonstrating that GL prevented palmitate-induced insulin resistance in myotubes.

### 2.5. GL Improved Palmitate-Induced Insulin Resistance via AMPK Activation in C2C12 Myotubes

**Figure 6:**
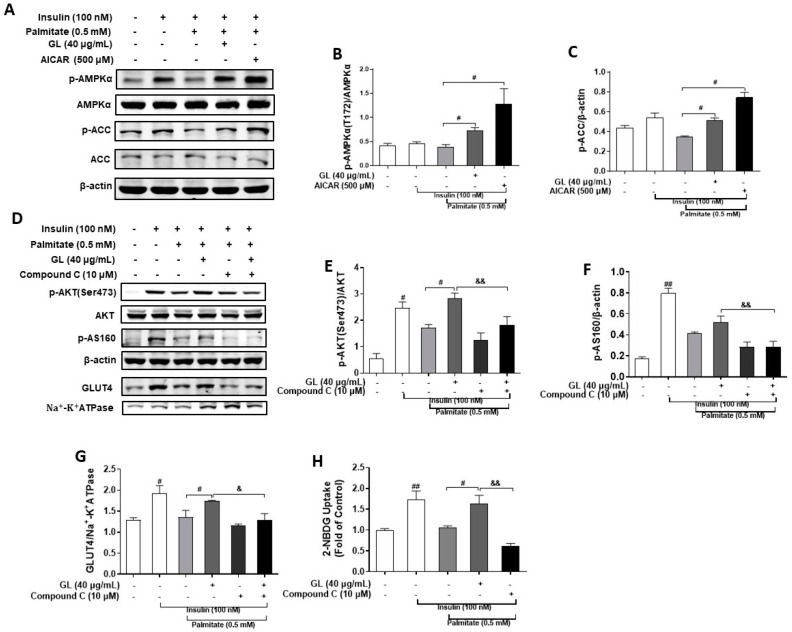
GL prevented palmitate-induced insulin resistance via AMPK signaling pathway in C2C12 myotubes. (**A**–**C**) GL activated AMPK pathway in palmitate-treated C2C12 myotubes. Western blotting analysis and quantification of the phosphorylation levels of AMPK and ACC (*n* = 3). (**D**–**F**) The increase in Akt phosphorylation and GLUT4 translocation by GL in insulin-resistant C2C12 myotubes was suppressed by compound C. Western blotting and quantification of phospho-AKT and phospho-AS160 (*n* = 3). Western blotting (**D**) and quantification (**G**) of GLUT4 in cell membrane proteins (*n* = 3). (**H**) Compound C mitigated the effect of GL on glucose uptake in insulin-resistant C2C12 myotubes. 2-NBDG assay of glucose uptake (*n* = 5). Values are presented as means ± SD. # and ## denote *p* < 0.05 and *p* < 0.01 compared to model (insulin+ palmitate) group, respectively; & and && denote *p* < 0.05 and *p* < 0.01 compared to GL group, respectively.

AMPK signaling is a key protective mechanism against palmitate-induced insulin resistance in myotubes. To explore whether AMPK involved the protective effects of GL, we detected the effect of GL on AMPK activation. As shown in Figure 6A–C, GL significantly upregulated phosphorylation of AMPK and ACC (*p* < 0.05), indicating that GL also significantly activated the AMPK pathway in palmitate-induced insulin-resistant myotubes. Then, we hypothesized that GL probably improved palmitate-induced insulin resistance in an AMPK-dependent mechanism. To test this hypothesis, compound C was used. Results showed that exposure of myotubes to palmitate led to insulin resistance, evidenced by decreased insulin-stimulated AKT phosphorylation and glucose uptake. GL treatment (40 μg/mL) effectively normalized insulin-mediated phosphorylation of AKT and AS160 (*p* < 0.05; Figure 6D–F) in myotubes exposed to palmitate. Co-treatment with AMPK inhibitor compound C diminished the effects of GL on AKT and AS160 phosphorylation, suggesting the possible involvement of AMPK in the regulation. Palmitate treatment impaired insulin-mediated GLUT4 translocation and glucose uptake. Co-treatment of myotubes with GL (40 μg/mL) improved insulin-mediated GLUT4 translocation and glucose uptake (*p* < 0.05; Figure 6D,G,H), whereas these effects were blocked by pretreatment with compound C (*p* < 0.05 or 0.01; Figure 6D,G,H). These results indicated that GL improved palmitate-induced insulin resistance via AMPK activation.

### 2.6. GC–MS Characterization

**Figure 7:**
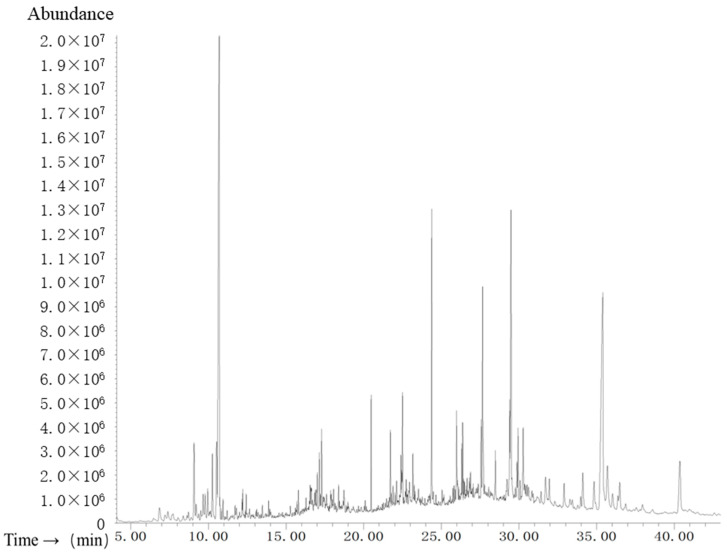
Total ion chromatogram of the lipophilic extract from ginkgo leaves.

The results of GC–MS analysis revealed the presence of various components in the GL (Figure 7 and Table S1). These compounds are presented in Supplementary Table S1. As the results showed, the major compounds detected in the GL were limonene, (4-Octyldodecyl)cyclopentan, 3-N-pentadecylphenol, phytol, vitamin E, γ-Sitosterol, cis-13-Docosenoamide, heptacosane, and 1-tricosene.

## 3. Discussion

*Ginkgo biloba* L. is one of the most popular medicinal plants in the world, with most sales coming from special extracts made from its leaves [15]. These extracts are enriched with terpene trilactones and flavonol glycosides and are standardized for their content [15]. However, other bioactive constituents in ginkgo leaves have received less attention. In the present study, the lipophilic extract from ginkgo leaves (GL) was found to have direct benefits on glucose metabolism. This extract was found to significantly enhance the glucose uptake in C2C12 myotubes under basal conditions, as well as the glucose uptake stimulated by insulin in insulin-resistant myotubes. These observed effects were found to be mediated by AMPK activation.

Muscles play a key role in regulating energy balance and are considered the most important tissue for glucose disposal [8,16]. Therefore, C2C12 myotubes were used to investigate the effect of GL on glucose metabolism in this study and it was found that GL significantly enhanced basal glucose uptake and glucose consumption. The most crucial step in controlling glucose uptake is the movement of GLUT4 from inside the cell vesicles to the plasma membrane [17]. Our measurements of GLUT4 levels in membrane proteins revealed a significant increase following GL treatment. This suggests that GL triggered the relocation of GLUT4 to the cell membrane, which in turn stimulated the basal glucose uptake.

The PI3K/AKT and AMPK pathways play a key role In regulation of glucose uptake in skeletal muscle [18,19]. AMPK is considered as a potential therapeutic target for treating metabolic syndrome [20]. The regulation of AMPK is critically dependent on the Thr172 site on its α subunit [7,20,21]. Once activated, AMPK has the ability to phosphorylate a range of kinases and other downstream proteins, thereby executing its diverse functions. For instance, AMPK can phosphorylate and suppress ACC, which in turn facilitates the transport of fatty acids and the subsequent β-oxidation [7]. The protein p38 MAPK, which operates downstream of AMPK, plays a role in the regulation of glucose uptake, a process that is dependent on AMPK [22,23,24]. AMPK also phosphorylates AS160, which ultimately promotes the translocation of GLUT4 from vesicles to the plasma membrane, stimulating glucose uptake [7,25]. In this study, we found that GL increased the phosphorylation of AMPK on Thr172 and its downstream targets ACC, p38 MAPK, and AS160 in a dose-dependent manner, indicating that this extract significantly activates the AMPK pathway. Based on these results, we hypothesized that GL promotes glucose uptake by activating the AMPK signaling pathway. To test this hypothesis, the AMPK inhibitor compound C and AMPKα1 siRNA were used to block AMPK activity in the present study. Results showed that compound C significantly blocked GL-induced activation of AMPK, p38 MAPK, and AS160, as well as GL-induced GLUT4 translocation and glucose uptake. In addition, AMPK siRNA interference significantly blocked GL-induced activation of AMPK, GLUT4 translocation, and glucose uptake. Thus, our data indicate that GL promotes GLUT4 translocation and subsequently stimulates glucose uptake via the AMPK-p38 MAPK-AS160 pathway. 

AMPK is regulated by various upstream kinases, such as CaMKKβ [14]. In this study, preincubation of GL with STO-609, a CaMKKβ inhibitor, abolished the GL-induced activation of the AMPK pathway, GLUT4 translocation, and glucose uptake. Overall, our results suggest that GL promotes glucose uptake in C2C12 myotubes by enhancing GLUT4 translocation to the plasma membrane via the CaMKKβ-AMPK-p38 MAPK-AS160 pathway.

Skeletal muscle is a key target tissue for insulin and plays a crucial role in maintaining body glucose metabolism. When skeletal muscle becomes resistant to insulin, its ability to take up glucose in response to insulin is significantly reduced. This leads to persistently high blood glucose levels and can ultimately result in metabolic disorders such as type 2 diabetes [5,6]. Insulin resistance in skeletal muscle is the primary problem, and addressing it can restore whole-body glucose homeostasis [7,8]. Therefore, promoting glucose uptake and insulin sensitivity in skeletal muscles plays a crucial role in preventing or reducing insulin resistance, hyperglycemia, and type 2 diabetes. 

In this study, GL was found to significantly stimulate basal glucose uptake in C2C12 myotubes, but its effect on insulin resistance, a condition often associated with impaired insulin signaling and reduced glucose uptake, remains unclear. Palmitate, a common dietary saturated free fatty acid, has been found to directly impair insulin signaling and induce insulin resistance in cultured myotubes and hepatocytes [26,27]. To evaluate the effects of GL on insulin resistance, palmitate was used to induce an insulin resistance model in C2C12 myotubes. We then treated GL with insulin-resistant C2C12 myotubes and examined the effects of GL on insulin signaling and insulin-induced glucose uptake. Results demonstrated that treatment myotubes exposed to palmitate with GL restored insulin signaling actions and reversed the inhibitory effects of palmitate on insulin-stimulated GLUT4 translocation and glucose uptake. This extract reversed the inhibitory effects of palmitate on insulin-stimulated PI3K (P110β) expression, phosphorylation of AKT and AS160, which consequently increased insulin-induced GLUT4 translocation and glucose uptake. These results indicate that GL improved palmitate-induced insulin resistance in C2C12 myotubes.

AMPK activation has been shown to enhance glucose uptake independent insulin as well as reduce insulin resistance in skeletal muscle [7,18,28,29]. GL was found to increase basal glucose uptake in C2C12 myotubes by activating AMPK. To test whether GL could improve insulin resistance through AMPK activation, we used compound C to block AMPK activity. Our results showed that pretreatment with compound C blocked the stimulatory effect of GL on insulin-stimulated GLUT4 translocation and glucose uptake. Additionally, the ameliorative effects of GL on insulin-stimulated phosphorylation of AKT and AS160 were abolished by compound C treatment in palmitate-treated cells. These findings suggest that GL improves palmitate-induced insulin resistance through the AMPK pathway.

## 4. Material and Methods

### 4.1. Chemicals and Reagents

Trypsin solutions, Dulbecco’s Modified Eagle’s Medium (DMEM), fetal bovine serum (FBS), antibiotic/antimycotic, and horse serum were purchased from GIBCO Life Technologies (Gaithersburg, MD, USA). We obtained 5-aminoimidazole-4-carboxamide ribonucleotide (AICAR) and Dimethyl sulfoxide (DMSO) from Sigma-Aldrich Chemical Co. (St. Louis, MO, USA). We used a Mem-PER Plus Membrane Protein Extraction Kit and 2-(N-[7-nitrobenz-2-oxa-1,3-diazol-4-yl] Amino)-2-deoxyglucose (2-NBDG) from Thermo (Sunnyvale, CA, USA). Protease inhibitor cocktail, phosphatase inhibitor cocktail, Compound C, and STO-609 were obtained from Sellbeck chemicals (Houston, TX, USA). Our siRNA was purchased from Shanghai GenePharma company (Shanghai, China). We utilized antibodies against phospho-AKT (Ser473), AMPKα (Thr172), phospho-AKT (Thr308), phospho-AS160 (Ser588), phospho-p38 MAPK (Thr180/Tyr182), phospho-ACC (Ser79), phosphoinositide 3-kinase (PI3K, P110β), AS160, AMPKα, and ACC from Cell Signaling Technology (Danvers, MA, USA); AKT, ATP1A1, p38 MAPK, and β-actin from Proteintech (Wuhan, China); and GLUT4 from Abcam (Cambridge, UK). Secondary antibodies and insulin were sourced from Yeasen Biotech (Shanghai, China). A glucose assay kit was procured from Shanghai Kexin Biotechnology Research Institute (Shanghai, China).

### 4.2. Preparation of Lipophilic Extract from Ginkgo Leaves

The ginkgo leaves were dried, pulverized, and sieved through a 20-mesh sieve to obtain a fine powder. The powders were extracted twice with petroleum ether (material-to-liquid ratio of 1:7) for 1.5 h each at 50 °C. The extraction solutions were merged, purified through filtration, and subsequently concentrated at 50 °C under diminished pressure to yield a concentrated solution. An equal volume of 4% NaOH-methanol solution and a small amount of tannic acid were added to the concentrated solution, and the saponification hydrolysis reaction was carried out at 50 °C for 3 h. After the reaction was completed, the upper petroleum ether phase was extracted, filtered, and dried to obtain the total nonsaponifiable lipid extract (GL).

The stock solutions of GL (20, 40 mg/mL) were prepared by dissolving this extract in DMSO and filtering it through a sterile syringe filter with a pore diameter of 0.22 μm. Prior to treatment, the stock solutions were further diluted to obtain various testing concentrations.

### 4.3. Chromatography–Mass Spectrometry (GC/MS)

The GL was subjected to analysis using gas chromatography coupled with mass spectrometry (GC–MS), utilizing an Agilent Technologies 7890B/5977B series GC–MS device (Santa Clara, CA, USA). The silica column employed was an Agilent DB-5, 30 m × 0.25 mm i.d., with a film thickness of 0.25 μm. The pyrolysis furnace was set at a temperature of 300 °C, with a pyrolysis duration of 10 s. The gas chromatography conditions were as follows: the initial column temperature was set at 50 °C, held for 5 min, then increased to 423 °C at a heating ramp of 10 °C/min, and maintained for 15 min. The injection was carried out in split mode with a split ratio of 20:1. The carrier gas was helium, operating in an isobaric mode at 6.0 kPa. The components were identified by comparing their mass spectra with those in the NIST08 libraries.

### 4.4. Palmitate Solution Preparation

Palmitate was first dissolved in pure ethanol and then diluted in DMEM that contained 2% BSA free of fatty acids to create a 10x concentrated palmitate stock solution. The control group received a similar volume of ethanol added to the BSA-DMEM solution. All solutions were filtered, aliquoted, and stored at 4 °C for subsequent use.

### 4.5. Cell Culture and Treatment

C2C12 mouse myoblasts were obtained from The National Center for Drug Screening (Shanghai, China). Myoblasts were cultured in high-glucose (~25 mM) DMEM supplemented with 10% (*v*/*v*) FBS, 100 U/mL streptomycin, and 100 U/mL penicillin, and maintained at 37 °C in a 5% CO_2_ atmosphere. The myoblasts were seeded onto culture plates at a density of 5 × 10^4^ cells/mL. After approximately 24 h, when the cells reached about 70% confluence, the medium was switched to DMEM supplemented with 2% (*v*/*v*) horse serum. This medium was replaced on days 2, 4, and 6 of culture. After 6–7 days, the differentiation of C2C12 mouse myoblasts into myotubes was complete.

To determine the effects of GL on glucose uptake, GLUT4 translocation, and AMPK pathway, myotubes were treated with various doses of GL (20 and 40 μg/mL) for 4 h, and glucose concentrations in the medium were measured using a glucose oxidase assay kit. Glucose consumption was then calculated by subtracting the post-treatment glucose concentration from the initial concentration in the culture medium.

To investigate the mechanism of stimulatory effect of GL on glucose uptake, an AMPK inhibitor compound C (15 µM), a CaMKKβ inhibitor STO-609 (15 µM), and AMPK siRNA (60 nM) were used. Differentiated myotubes were pretreated with compound C or STO-609 for 1 h before treatment with GL (40 μg/mL) for 4 h. Differentiated myotubes were transfected with normal control or AMPK siRNA (60 nM) for 48 h followed by exposure to GL for 4 h.

To test the effect of GL on palmitate-induced insulin resistance in C2C12 myotubes, differentiated myotubes were exposed to palmitate (0.5 mM) with GL (40 μg/mL) for 18 h. In parallel, myotubes treated with an equivalent volume of DMSO but without palmitate and GL were used as the normal control, and those treated with an equivalent volume of DMSO and palmitate without GL were used as the model control. If the cells needed to be exposed to insulin, the insulin (100 nM) was added into medium 30 min before cells were harvested.

To investigate the mechanism of action of GL during palmitate-induced insulin resistance, compound C (10 μM) and AMPK siRNA (60 nM) were used. Differentiated myotubes were incubated with compound C (10 µM) for 1 h followed by treated with palmitate (0.5 mM) and GL (40 μg/mL) for 18 h, and then stimulated by insulin (100 nM) for 30 min. Differentiated myotubes were treated with normal control or AMPK siRNA (60 nM) for 48 h followed by incubated palmitate (0.5 mM) and GL (40 μg/mL) for 18 h, and then stimulated by insulin (100 nM) for 30 min. In parallel, myotubes treated with an equivalent volume of DMSO but without palmitate, insulin, and GL were used as the normal control, and those treated with an equivalent volume of DMSO, insulin, and palmitate without GL were used as the insulin resistance model control.

### 4.6. MTT Assay

The effect of GL on cell viability was measured using a 3-(4,5-dimethyl-2-thia-zolyl)-2,5-diphenyl-2-H-tetrazoliumbromide (MTT) assay. 

C2C12 mouse myoblasts were initially cultured in 96-well plates and subsequently differentiated into myotubes. Post-differentiation, the myotubes were incubated in DMEM containing 0.2% BSA for a duration of 6 h. The culture medium was then replaced with DMEM containing 0.2% BSA and concentrations ranging from 0.6 to 80 μg/mL GL or 100 nM insulin, and incubated for 24 h. Following this incubation period, each well was treated with 20 μL of 3 mg/mL MTT and incubated for an additional 2.5 h at 37 °C. Subsequently, 200 μL of DMSO was added to each well and the plate was agitated until the MTT formazan crystals were completely dissolved. The absorbance of each well was then measured at 490 nm using a microplate spectrophotometer, and cell viability was calculated using the following formula:

$$Cell\;Viability=\frac{{OD}_{Sample}}{{OD}_{Control}}\;\times \;100\%$$

### 4.7. Glucose Consumption Assay 

Glucose consumption was quantified from the culture media using a glucose assay kit. Following differentiation, C2C12 myotubes were incubated overnight and subsequently exposed to 5, 10, 20, and 40 μg/mL GL for periods of 24, 48, and 72 h. Post-treatment, the glucose levels in the medium were assessed using a commercially glucose assay kit, adhering to the manufacturer’s guidelines. The calculation of the glucose consumption was performed using the following equation:

$$Glucose\;Consumption\;(mg/L)=4500-\frac{{OD}_{Sample}-{OD}_{Water}}{{OD}_{Standard}-{OD}_{Water}}\;\times \;5.55\;\times \;180$$

### 4.8. Transfection with Small-Interfering RNA (siRNA)

C2C12 myotubes were transfected with AMPKα1 siRNA and negative control siRNA (60 nM; Shanghai GenePharma, Shanghai, China) using LipofectamineTM 3000 Reagent (Invitrogen, Carlsbad, CA, USA) in DMEM medium, following the manufacturer’s protocol. After 48 h of transfection, the efficiency was evaluated by performing Western blotting against the AMPKα1 antibody.

AMPKα1 siRNA: 

Sense (5′-3′) UUUGAAAGACCAAAGUCGGCU.

Antisense (5′-3′) CCGACUUUGGUCUUUCAAACA.

Negative control siRNA: 

Sense (5′-3′) UUCUCCGAACGUGUCACGUTT.

Antisense (5′-3′) ACGUGACACGUUCGGAGAATT.

### 4.9. 2-NBDG Uptake Assay

Cell glucose uptake was determined by measuring 2-NBDG uptake according to the following procedure. Differentiated C2C12 mouse myotubes were cultured in the black 96-well plates and treated with different conditions for a range of time periods. One hour prior to the harvest, the cells underwent two washes with phosphate-buffered saline (PBS) that had been sterilized and prewarmed to 37 °C. Following this, the medium was replaced with glucose-free DMEM, supplemented with 0.2% BSA. After an hour, the cells were washed once with sterilized PBS and warmed to 37 °C. They were then incubated for 30 min in the same medium, now containing 80 μM 2-NBDG. Subsequent to this incubation, the cells were washed once more with PBS sterilized and warmed to 37 °C. The fluorescence intensity of each well was then measured (with 485 nm excitation and 520 nm emission). The glucose uptake of the cells was calculated using the following formula:

$$Glucose\;Uptake=\frac{{FI}_{Sample}-{FI}_{Blank}}{{FI}_{Control}-{FI}_{Blank}}$$

### 4.10. Western Blotting Analysis

Following treatment, the cells were subjected to two washes with PBS that had been chilled to 4 °C before being harvested in a lysis buffer for radioimmunoprecipitation assay (containing 150 mmol/L sodium chloride, 1.0% Triton X-100, 0.5% sodium deoxycholate, 0.1% sodium dodecyl sulfate, and 50 mmol/L Tris at pH 8.0), which also included a protease inhibitor cocktail and a phosphatase inhibitor cocktail. The extraction of cell membrane proteins was performed using a Mem-PERa Plus Membrane Protein Extraction Kit, adhering to the manufacturer’s protocol. The protein concentrations were ascertained using a Bicinchoninic acid Protein Assay Kit, again following the protocol of the manufacturer. Proteins, in equal quantities, were separated on 10% sodium dodecyl sulfate-polyacrylamide gels and then transferred onto nitrocellulose membranes. These membranes were incubated overnight at 4 °C with primary antibodies. After three washes in Tris-buffered saline (containing 0.1% Tween-20), the membranes were incubated for 1 h with secondary antibodies at room temperature. Finally, the blots were washed and visualized using an Odyssey CLx Imaging System (LI-COR, Lincoln, NE, USA), and the resulting images were analyzed using Image-Pro Plus Software 6.0 (Media Cybernetics, Rockville, MD, USA).

### 4.11. Statistical Analysis

Data are presented as mean ± standard deviation (SD). One-way ANOVA followed by Dunnett’s tests were used for statistical analysis using SPSS (IBM, Armonk, NY, USA). *p* values of <0.05 and <0.01 were considered significant and extremely significant, respectively.

## 5. Conclusions 

This study shows that GL enhances basal glucose uptake and protects against palmitate-induced insulin resistance by activating AMPK in C2C12 myotubes. These results suggest that GL has the potential to lower blood glucose levels and prevent insulin resistance, indicating that the lipophilic extract from ginkgo leaves could be a promising antidiabetic agent.

## Data Availability

The data are included in the figures and tables of this manuscript.

## Supplementary Materials

The following supporting information can be downloaded at: https://www.mdpi.com/article/10.3390/molecules29071605/s1, Table S1. GC–MS analysis revealed the presence of phytochemical components in the lipophilic extract from ginkgo leaves.

## Abbreviations

2-NBDG	2-(N-(7-Nitrobenz-2-oxa-1,3-diazol-4-yl) Amino)-2-deoxyglucose.
ACC	Acetyl-CoA carboxylase.
AICAR	5-aminoimidazole-4-carboxamide ribonucleotide.
AMPK	5′-adenosine monophosphate-activated protein kinase.
AS160	AKT substrate of 160 kDa.
CaMKKβ	Calcium/calmodulin-dependent protein kinase kinase β.
DMEM	Dulbecco’s Modified Eagle Medium.
FBS	Fetal bovine serum.
GL	The lipophilic extract from *Ginkgo biloba* L. leaves.
GLUT4	Glucose transport 4.
GL	The lipophilic extract from *Ginkgo biloba* L. leaves.
p38 MAPK	p38 mitogen-activated protein kinase.
PI3K	Phosphoinositide 3-kinase.
PKB/AKT	Protein kinase B.
