# Supplementary material for: The Lipophilic Extract from Ginkgo biloba L. Leaves Promotes Glucose Uptake and Alleviates Palmitate-Induced Insulin Resistance in C2C12 Myotubes
Source: Molecules. 2024 Apr 3;29(7):1605. doi: 10.3390/molecules29071605 (PMC11013672; doi:10.3390/molecules29071605)
Supplement: Supplementary file 1 [file molecules-29-01605-s001.zip › molecules-2889663-supplementary.pdf]

Table S1 GC-MS analysis revealed the presence of phytochemical components in the lipophilic extract from ginkgo leaves.

PK	RT (min)	Area (%)	Compound	CAS	Qual
12	6.8588	0.3068	p-Xylene	000106-42-3	97
21	9.0884	0.8211	Cyclohexene, 4-ethenyl-1,4-dimethyl-	001743-61-9	94
26	9.6608	0.303	cis-2,6-Dimethyl-2,6-octadiene	002492-22-0	87
28	9.9621	0.3439	cis-2,6-Dimethyl-2,6-octadiene	002492-22-0	91
33	10.5346	0.9223	Cyclohexene, 1-methyl-4-(1-methylethyl)-, (R)-	001195-31-9	95
34	10.7053	6.8226	Limonene	000138-86-3	94
36	10.9463	0.1483	1,3,6-Octatriene, 3,7-dimethyl-	013877-91-3	97
46	11.8101	0.1636	Benzene, 1-methyl-4-(1-methylethenyl)-	001195-32-0	91
52	12.2218	0.2107	1,3,8-p-Menthatriene	021195-59-5	94
55	12.4428	0.1576	2,4,6-Octatriene, 2,6-dimethyl-, (E,Z)-	007216-56-0	97
58	12.6537	0.101	2,4,6-Octatriene, 2,6-dimethyl-	000673-84-7	97
69	13.4772	0.1475	Benzene, 1-methyl-3-(1-methylethyl)-	000535-77-3	91
71	13.6781	0.1109	Cyclohexanone, 2-methyl-5-(1-methylethenyl)-, trans-	005948-04-9	93
74	13.8889	0.1102	5-Isopropenyl-2-methylcyclopent-1-enecarboxaldehyde	1000190-36-8	97

104	16.2993	0.15	Azulene, 1,2,3,5,6,7,8,8a-octahydro-1,4-dimethyl-7-(1-methylethenyl)-, [1S-(1.alpha.,7.alpha.,8a.beta.)]-	003691-11-0	83
105	16.3696	0.1222	1H-Cycloprop[e]azulene, 1a,2,3,5,6,7,7a,7b-octahydro-1,1,4,7-tetramethyl-, [1aR-(1a.alpha.,7.alpha.,7a.beta.,7b.alpha.)]-	021747-46-6	93
108	16.5102	0.1001	Humulen-(v1)	1000159-39-4	86
109	16.5604	0.3125	.alpha.-Farnesene	000502-61-4	87
114	16.8416	0.2326	Cycloheptane, 4-methylene-1-methyl-2-(2-methyl-1-propen-1-yl)-1-vinyl-	1000159-38-5	89
115	16.9119	0.2127	Azulene, 1,2,3,5,6,7,8,8a-octahydro-1,4-dimethyl-7-(1-methylethenyl)-, [1S-(1.alpha.,7.alpha.,8a.beta.)]-	003691-11-0	93
118	17.2232	0.1909	Naphthalene, decahydro-4a-methyl-1-methylene-7-(1-methylethenyl)-, [4aR-(4a.alpha.,7.alpha.,8a.beta.)]-	017066-67-0	94
120	17.4141	0.2781	Spiro[4.5]dec-7-ene, 1,8-dimethyl-4-(1-methylethenyl)-, [1S-(1.alpha.,4.beta.,5.alpha.)]-	024048-44-0	91
121	17.4643	0.257	1H-Cycloprop[e]azulene, 1a,2,3,5,6,7,7a,7b-octahydro-1,1,4,7-tetramethyl-, [1aR-(1a.alpha.,7.alpha.,7a.beta.,7b.alpha.)]-	021747-46-6	94
122	17.5547	0.1372	.beta.-Humulene	000116-04-1	90
128	17.876	0.222	.alpha.-Farnesene	000502-61-4	95

129	17.9162	0.1768	Cyclohexane, 1-ethenyl-1-methyl-2-(1-methylethenyl)-4-(1-methylethylidene)-	003242-08-8	89
130	17.9765	0.1379	.beta.-Humulene	000116-04-1	93
132	18.0668	0.2765	Cyclohexane, 1-ethenyl-1-methyl-2-(1-methylethenyl)-4-(1-methylethylidene)-	003242-08-8	89
133	18.1572	0.1983	.beta.-Humulene	000116-04-1	94
135	18.3882	0.4239	Spiro[4.5]dec-7-ene, 1,8-dimethyl-4-(1-methylethenyl)-, [1S-(1.alpha.,4.beta.,5.alpha.)]-	024048-44-0	89
136	18.4485	0.1324	Azulene, 1,2,3,5,6,7,8,8a-octahydro-1,4-dimethyl-7-(1-methylethenyl)-, [1S-(1.alpha.,7.alpha.,8a.beta.)]-	003691-11-0	89
137	18.4987	0.1916	1,3,3-Trimethyl-2-hydroxymethyl-3,3-dimethyl-4-(3-methylbut-2-enyl)-cyclohexene	1000144-10-7	81
144	19.031	0.164	Hexadecane	000544-76-3	91
146	19.2419	0.2039	4,6,6-Trimethyl-2-(3-methylbuta-1,3-dienyl)-3-oxatricyclo[5.1.0.0(2,4)]octane	1000190-22-2	90
147	19.3323	0.2907	1H-Cycloprop[e]azulene, decahydro-1,1,7-trimethyl-4-methylene-, [1aR-(1a.alpha.,4a.beta.,7.alpha.,7a.beta.,7b.alpha.)]-	025246-27-9	89

154	20.0453	0.1483	4,6,6-Trimethyl-2-(3-methylbuta-1,3-dienyl)-3-oxatricyclo[5.1.0.0(2,4)]octane	1000190-22-2	91
156	20.1759	0.1431	Hexadecane	000544-76-3	95
159	20.3768	0.1146	4,6,6-Trimethyl-2-(3-methylbuta-1,3-dienyl)-3-oxatricyclo[5.1.0.0(2,4)]octane	1000190-22-2	90
161	20.4872	0.6722	1-Heptene, 2-isohexyl-6-methyl-	033717-93-0	90
164	20.7785	0.1543	.beta.-Humulene	000116-04-1	93
171	21.2003	0.1213	1-Octadecene	000112-88-9	91
172	21.2706	0.1847	Octadecane	000593-45-3	95
178	21.7225	0.4985	2-Pentadecanone, 6,10,14-trimethyl-	000502-69-2	91
180	21.8932	0.2652	Oxirane, 2,2-dimethyl-3-(3,7,12,16,20-pentamethyl-3,7,11,15,19-heneicosapentaenyl)-, (all-E)-	007200-26-2	86
186	22.2548	0.1304	1-Nonadecene	018435-45-5	93
187	22.3251	0.2564	.beta.-Humulene	000116-04-1	89
188	22.4054	0.4781	Geranylgeraniol	024034-73-9	91
191	22.5762	0.1596	Naphthalene, 1,2,3,4,4a,5,6,8a-octahydro-4a,8-dimethyl-2-(1-methylethenyl)-, [2R-(2.alpha.,4a.alpha.,8a.beta.)]-	000473-13-2	91

194	22.7871	0.3603	1,3,6,10-Cyclotetradecatetraene, 3,7,11-trimethyl-14-(1-methylethyl)-, [S-(E,Z,E,E)]-	001898-13-1	92
196	22.9578	0.3871	1,3,6,10-Cyclotetradecatetraene, 3,7,11-trimethyl-14-(1-methylethyl)-, [S-(E,Z,E,E)]-	001898-13-1	83
197	23.0382	0.2165	1,3,6,10-Cyclotetradecatetraene, 3,7,11-trimethyl-14-(1-methylethyl)-, [S-(E,Z,E,E)]-	001898-13-1	87
200	23.3194	0.2141	Eicosane	000112-95-8	90
201	23.3595	0.1201	Naphthalene, 1,2,3,4,4a,5,6,8a-octahydro-4a,8-dimethyl-2-(1-methylethenyl)-, [2R-(2.alpha.,4a.alpha.,8a.beta.)]-	000473-13-2	95
204	23.5905	0.1154	1,3,6,10-Cyclotetradecatetraene, 3,7,11-trimethyl-14-(1-methylethyl)-, [S-(E,Z,E,E)]-	001898-13-1	89
208	23.8215	0.118	1,3,6,10-Cyclotetradecatetraene, 3,7,11-trimethyl-14-(1-methylethyl)-, [S-(E,Z,E,E)]-	001898-13-1	83
209	23.8818	0.118	Naphthalene, 1,2,3,4,4a,5,6,8a-octahydro-4a,8-dimethyl-2-(1-methylethenyl)-, [2R-(2.alpha.,4a.alpha.,8a.beta.)]-	000473-13-2	86
213	24.2132	0.3036	.beta.-Humulene	000116-04-1	83
214	24.2634	0.1985	Nonadecane	000629-92-5	86

215	24.3839	2.1825	Phytol	000150-86-7	95
217	24.5647	0.1448	Naphthalene, 1,2,3,4,4a,5,6,8a-octahydro-4a,8-dimethyl-2-(1-methylethenyl)-, [2R-(2.alpha.,4a.alpha.,8a.beta.)]-	000473-13-2	93
219	24.7154	0.1134	4,8-Methanoazulen-9-ol, decahydro-2,2,4,8-tetramethyl-, stereoisomer	004586-22-5	90
220	24.7555	0.1068	Naphthalene, 1,2,3,4,4a,5,6,8a-octahydro-4a,8-dimethyl-2-(1-methylethenyl)-, [2R-(2.alpha.,4a.alpha.,8a.beta.)]-	000473-13-2	86
221	24.8158	0.2902	Cycloheptane, 4-methylene-1-methyl-2-(2-methyl-1-propen-1-yl)-1-vinyl-	1000159-38-5	80
222	24.9463	0.1979	Naphthalene, 1,2,3,4,4a,5,6,8a-octahydro-4a,8-dimethyl-2-(1-methylethenyl)-, [2R-(2.alpha.,4a.alpha.,8a.beta.)]-	000473-13-2	93
224	25.1271	0.1659	1-Nonadecene	018435-45-5	90
225	25.1673	0.2649	Tetracosane	000646-31-1	96
228	25.4184	0.1598	(1R,2S,8R,8Ar)-8-hydroxy-1-(2-hydroxyethyl)-1,2,5,5-tetramethyl-trans-decalin	1000298-98-3	83
231	25.579	0.203	Cycloheptane, 4-methylene-1-methyl-2-(2-methyl-1-propen-1-yl)-1-vinyl-	1000159-38-5	83
235	25.8201	0.2941	Farnesol isomer a	1000108-92-4	89
237	25.9808	6.1472	3-N-Pentadecylphenol	000501-24-6	80
240	26.3222	0.6337	1,6,10,14,18,22-Tetracosahexaen-3-ol, 2,6,10,15,19,23-hexamethyl-, (all-E)-	054159-46-5	80

241	26.3825	0.5969	Squalene	007683-64-9	89
242	26.4428	0.2802	1,6,10,14,18,22-Tetracosahexaen-3-ol, 2,6,10,15,19,23-hexamethyl-, (all-E)-	054159-46-5	80
243	26.493	0.2676	4,8,12,16-Tetramethylheptadecan-4-olide	096168-15-9	95
245	26.6637	0.4136	2,3-Dimethoxy-5-methyl-6-dekaisoprenyl-chinon	060684-33-5	86
248	26.8846	0.3189	Tetracosane	000646-31-1	99
249	26.9449	0.1929	.beta.-Humulene	000116-04-1	91
252	27.1859	0.4198	Cycloheptane, 4-methylene-1-methyl-2-(2-methyl-1-propen-1-yl)-1-vinyl-	1000159-38-5	90
253	27.2462	0.3708	Cycloheptane, 4-methylene-1-methyl-2-(2-methyl-1-propen-1-yl)-1-vinyl-	1000159-38-5	89
259	27.7785	0.438	Curan-17-oic acid, 2,16-didehydro-20-hydroxy-19-oxo-, methyl ester	056053-15-7	91
260	27.8789	0.1997	Curan-17-oic acid, 2,16-didehydro-20-hydroxy-19-oxo-, methyl ester	056053-15-7	91
261	27.9191	0.1261	Cycloheptane, 4-methylene-1-methyl-2-(2-methyl-1-propen-1-yl)-1-vinyl-	1000159-38-5	83
262	27.9593	0.283	Cycloheptane, 4-methylene-1-methyl-2-(2-methyl-1-propen-1-yl)-1-vinyl-	1000159-38-5	80
267	28.4815	0.9067	Hexacosane	000630-01-3	98
269	28.6522	0.2009	Cycloheptane, 4-methylene-1-methyl-2-(2-methyl-1-propen-1-yl)-1-vinyl-	1000159-38-5	80
270	28.7526	0.2622	Cycloheptane, 4-methylene-1-methyl-2-(2-methyl-1-propen-1-yl)-1-vinyl-	1000159-38-5	83
276	29.1845	0.2102	Curan-17-oic acid, 2,16-didehydro-20-hydroxy-19-oxo-, methyl ester	056053-15-7	91
278	29.4054	1.094	Heptacosane	000593-49-7	98

284	29.9478	0.7882	Oxirane, 2,2-dimethyl-3-(3,7,12,16,20-pentamethyl-3,7,11,15,19-heneicosapentaenyl)-, (all-E)-	007200-26-2	90
285	30.0783	0.4447	Curan-17-oic acid, 2,16-didehydro-20-hydroxy-19-oxo-, methyl ester	056053-15-7	92
286	30.1587	0.1918	Curan-17-oic acid, 2,16-didehydro-20-hydroxy-19-oxo-, methyl ester	056053-15-7	91
287	30.2692	1.1737	cis-13-Docosenoamide	000112-84-5	96
289	30.4399	0.328	Nonacosane	000630-03-5	97
293	30.9119	0.3018	1-Hexadecyne	000629-74-3	83
297	31.4241	0.4254	Curan-17-oic acid, 2,16-didehydro-20-hydroxy-19-oxo-, methyl ester	056053-15-7	90
299	31.7153	1.0387	1-Tricosene	018835-32-0	95
301	31.9564	0.648	2-Heptacosanone	007796-19-2	93
302	32.0769	0.4054	Curan-17-oic acid, 2,16-didehydro-20-hydroxy-19-oxo-, methyl ester	056053-15-7	91
303	32.2978	0.5728	Cholestane, 3-(methoxymethoxy)-, (3.beta.,5.alpha.)-	004707-81-7	90
305	32.6292	0.2037	Cyclohexane, 1-ethenyl-1-methyl-2,4-bis(1-methylethenyl)-, [1S-(1.alpha.,2.beta.,4.beta.)]-	000515-13-9	86
306	32.6795	0.1681	Cyclohexane, 1-ethenyl-1-methyl-2,4-bis(1-methylethenyl)-, [1S-(1.alpha.,2.beta.,4.beta.)]-	000515-13-9	86
307	32.81	0.1308	Curan-17-oic acid, 2,16-didehydro-20-hydroxy-19-oxo-, methyl ester	056053-15-7	91

309	33.282	0.5147	Octacosyl acetate	018206-97-8	90
312	33.7842	0.3172	1H-Benzocyclohepten-7-ol, 2,3,4,4a,5,6,7,8-octahydro-1,1,4a,7-tetramethyl-, cis-	006892-80-4	84
313	33.975	0.3713	1,19-Eicosadiene	014811-95-1	93
314	34.1156	0.8025	.gamma.-Tocopherol	007616-22-0	97
322	35.7024	1.4978	Vitamin E	000059-02-9	99
323	36.0238	0.7323	Vitamin E	000059-02-9	96
324	36.3753	0.3595	Geranylgeraniol	024034-73-9	93
325	36.4958	0.746	2,6,10,14,18,22-Tetracosahexaene, 2,6,10,15,19,23-hexamethyl-, (all-E)-	000111-02-4	91
327	36.8775	0.3206	Curan-17-oic acid, 2,16-didehydro-20-hydroxy-19-oxo-, methyl ester	056053-15-7	92
330	37.3997	0.2308	Curan-17-oic acid, 2,16-didehydro-20-hydroxy-19-oxo-, methyl ester	056053-15-7	91
331	37.5604	0.2726	Curan-17-oic acid, 2,16-didehydro-20-hydroxy-19-oxo-, methyl ester	056053-15-7	91
332	37.8014	0.1765	Curan-17-oic acid, 2,16-didehydro-20-hydroxy-19-oxo-, methyl ester	056053-15-7	91
333	37.9621	0.4284	Campesterol	000474-62-4	97
337	38.6049	0.266	Stigmasterol	000083-48-7	91
347	39.4384	0.1333	Curan-17-oic acid, 2,16-didehydro-20-hydroxy-19-oxo-, methyl ester	056053-15-7	91
350	39.9908	0.1177	Curan-17-oic acid, 2,16-didehydro-20-hydroxy-19-oxo-, methyl ester	056053-15-7	91

352	40.3624	1.4133	.gamma.-Sitosterol	000083-47-6	99

RT: retention time; Qual: quality of resemblance
